# Immune Protection of Nonhuman Primates against Ebola Virus with Single Low-Dose Adenovirus Vectors Encoding Modified GPs

**DOI:** 10.1371/journal.pmed.0030177

**Published:** 2006-05-16

**Authors:** Nancy J Sullivan, Thomas W Geisbert, Joan B Geisbert, Devon J Shedlock, Ling Xu, Laurie Lamoreaux, Jerome H. H. V Custers, Paul M Popernack, Zhi-Yong Yang, Maria G Pau, Mario Roederer, Richard A Koup, Jaap Goudsmit, Peter B Jahrling, Gary J Nabel

**Affiliations:** 1Vaccine Research Center, National Institute of Allergy and Infectious Diseases, National Institutes of Health, Bethesda, Maryland, United States of America; **2**United States Army Medical Research Institute of Infectious Diseases, Fort Detrick, Maryland, United States of America; **3**Crucell Holland B.V., Leiden, Netherlands; **4**Integrated Research Facility, National Institute of Allergy and Infectious Diseases, National Institutes of Health, Bethesda, Maryland, United States of America; Mount Sinai School of MedicineUnited States of America

## Abstract

**Background:**

Ebola virus causes a hemorrhagic fever syndrome that is associated with high mortality in humans. In the absence of effective therapies for Ebola virus infection, the development of a vaccine becomes an important strategy to contain outbreaks. Immunization with DNA and/or replication-defective adenoviral vectors (rAd) encoding the Ebola glycoprotein (GP) and nucleoprotein (NP) has been previously shown to confer specific protective immunity in nonhuman primates. GP can exert cytopathic effects on transfected cells in vitro, and multiple GP forms have been identified in nature, raising the question of which would be optimal for a human vaccine.

**Methods and Findings:**

To address this question, we have explored the efficacy of mutant GPs from multiple Ebola virus strains with reduced in vitro cytopathicity and analyzed their protective effects in the primate challenge model, with or without NP. Deletion of the GP transmembrane domain eliminated in vitro cytopathicity but reduced its protective efficacy by at least one order of magnitude. In contrast, a point mutation was identified that abolished this cytopathicity but retained immunogenicity and conferred immune protection in the absence of NP. The minimal effective rAd dose was established at 10^10^ particles, two logs lower than that used previously.

**Conclusions:**

Expression of specific GPs alone vectored by rAd are sufficient to confer protection against lethal challenge in a relevant nonhuman primate model. Elimination of NP from the vaccine and dose reductions to 10^10^ rAd particles do not diminish protection and simplify the vaccine, providing the basis for selection of a human vaccine candidate.

## Abstract 2

A simplified Ebola vaccine that consists of a modified GP protein (which is well-tolerated by human cells even at high concentrations) in a replication-defective adenoviral vector protects macaques.

## Editors' Summary

**Background.:**

Humans who get infected with Ebola virus develop an illness called Ebola hemorrhagic fever (EHV), which is one of the most deadly viral diseases known; 50%–90% of all ill patients die, and there is no available treatment for EHV. Scientists think that the occasional outbreaks of the disease occur because the virus “jumps” from an infected animal to a person (a rare event) and then is transmitted between people by direct contact with infected blood or other body fluids or parts. Several strains or variants of the Ebola virus exist. Most outbreaks have been caused either by the Zaire strain or by the Sudan/Gulu strain (so-called because that is where the particular virus was first isolated). Scientists are working on a vaccine against Ebola that could be given to people before they get infected and then protect them when they come in contact with the virus. A number of candidate vaccines have been developed and tested in animals.

**Why Was This Study Done?:**

The researchers who did this study are working on a vaccine that consists of two particular parts of the virus. One part is called GP (which stands for glycoprotein) and is from the outer coat of the virus; the other, NP (nucleoprotein), is from its inside. Without the rest of the virus, GP and NP cannot cause EBV. However, the hope is that giving these parts of the virus to an individual can educate their immune system to build a response against GP and NP, which would then recognize the virus should the vaccinated person become infected with the whole virus, and destroy it before it can cause disease. To get the GP and NP parts into the body so that they can cause a strong immune response (which is what effective vaccines do), the researchers used a manmade version of another, harmless virus called recombinant adenovirus 5 (or rAd5) to carry the NP and GP. The researchers have shown previously that this strategy for introducing a vaccine works in animals. The vaccine—i.e., the combination of the rAd5 virus and the two Ebola virus parts—can protect animals against subsequent infection with real Ebola virus that would otherwise kill them. However, during these earlier studies, the researchers had noticed that the GP part, when present at high levels, seemed to make human cells sick. They had not seen any similar problems in the experimental animals, but to be on the safe side they decided to see whether they could change the GP part so that it would still be effective as a vaccine but no longer make human cells sick.

**What Did the Researchers Do and Find?:**

They changed the GP part of the vaccine in different ways so that it would no longer make human cells sick and then tested whether the resulting vaccines (combined with the original NP part and the Ad5 virus) could still protect monkeys from EHF after they were infected with Ebola virus. They found that some of the new GP versions made the vaccine less effective, but others did what they had hoped for; namely, they gave the same level of protection as when the original GP part was present. While doing these experiments, the researchers also found that the NP component seemed unnecessary and in some cases even weakened the vaccine's effect.

**What Do These Findings Mean?:**

The researchers have now developed a simplified vaccine against Ebola virus that is effective in monkeys. This vaccine consists of only a modified GP component (which is well tolerated by human cells even at high concentrations) and the rAd5 component. This vaccine is not the only candidate currently being developed against Ebola, but it seems likely that it is one of a few that will be tested in human volunteers in the near future. The initial clinical trials will test whether the vaccine is safe in humans, and whether it can cause the immune system to produce an immune response that is specific for the Ebola virus. Assuming that the outcomes of these trials are positive, the next question is whether the vaccine can protect humans against Ebola disease. Because Ebola is so dangerous and outbreaks are relatively rare, the vaccine will likely be tested only during an actual outbreak. At that time, an experimental vaccine might be given to people at immediate risk of becoming infected, especially health-care workers who, because they take care of infected patients, are themselves at very high risk of becoming infected. In addition to trials in humans, the scientists will also explore whether this vaccine, which was developed based on the GP component of the Zaire strain, can protect monkeys against infections with other strains of the Ebola virus.

**Additional Information.:**

Please access these Web sites via the online version of this summary at http://dx.doi.org/10.1371/journal.pmed.0030177.

• World Health Organization

• MedlinePlus Medical Encyclopedia

• US Centers for Disease Control and Prevention

• Wikipedia (note: Wikipedia is a free Internet encyclopedia that anyone can edit)

## Introduction

Epidemics of Ebola virus hemorrhagic fever result in a fatal illness in the majority of patients who become infected, with case-fatality rates nearing 90%. Studies of patients who survive infection indicate that components of the adaptive immune system are activated in infected survivors [1], suggesting that vaccination of target populations could significantly reduce mortality from exposure to Ebola virus. We previously described a vaccine that elicits strong adaptive immune responses and uniformly protects nonhuman primates from a high-dose challenge with Zaire ebolavirus (ZEBOV) [2,3]. A single immunization with vectors derived from adenovirus type 5 (5 Ad5) containing Ebola glycoprotein (GP) and nucleoprotein (NP) genes elicited protective immune responses within 1 mo. The strength of rAd5 vectors for use as vaccines derives from their ability to elicit rapid immunity due to high insert expression levels and natural targeting of Ad5 to dendritic cells, which may enhance antigen presentation. Moreover, the use of replication-defective viral vectors for gene delivery is desirable from a safety perspective for products that will be developed for human use.

Beyond considerations of vector choice, selection of the target viral antigen(s) must incorporate determination of relative magnitudes of protective immunity elicited by different inserts, and breadth of protection across virus subtypes. Ebola virus comprises four species, ZEBOV, Sudan ebolavirus (SEBOV), Ivory Coast, and Reston, with ZEBOV and SEBOV contributing the highest mortality in natural outbreaks. Therefore, the Zaire and Sudan (Gulu) ebolaviruses were selected for studies in which we sought to provide a basis for selection of a human vaccine candidate by identifying the optimal vaccine composition with respect to antigen strength and immune protection in nonhuman primates. Ebola NP was included in the vaccine because it is highly conserved among Ebola subtypes and has been found to elicit strong cellular immune responses in animal models [4,5].

## Methods

### Vector Construction and Transfections

The reagents used in this study were produced by Crucell (Leiden, Netherlands) following the Organization for Economic Cooperation and Development Principles of Good Laboratory Practice as guidance, and using methods similar to and designed for easy scale-up to GMP clinical-grade preparation. The GP point mutant vectors are currently in preparation for clinical trials. E1/E3-deleted, replication-incompetent Ad5 vectors were generated in PER.C6 cells [6] using a pBR322-based adaptor plasmid pAdApt together with the cosmid pWE.Ad.AfllII-rITRΔE3, essentially as described elsewhere [7]. The adaptor plasmid contained the left portion of the Ad5 genome (nucleotides 1–454), followed by transcriptional control elements and the adaptor Ad5 DNA region (nucleotides 3511–6095 in Ad5). Ebola GP-encoding genes were cloned into the expression cassette in the adaptor plasmids under transcriptional control of the human full-length immediate-early CMV promoter and the SV40 polyadenylation signal.

Adenoviruses containing Ebola GP, GPΔTM, and point mutations were generated by cotransfection of linearized pAdApt-Ebola GP plasmids together with the linearized cosmid pWE.Ad.AfllII-rITRΔE3 containing the right portion of the Ad5 genome to PER.C6 cells using Lipofectamine (Invitrogen, Carlsbad, California, United States). PER.C6 cells were cultured in DMEM supplemented with 10% fetal bovine serum (GIBCO, San Diego, California, United States) and incubated at 37 °C in a humidified atmosphere at 10% CO_2_. Homologous recombination led to the generation of rAd5-Ebola GP viruses. Adenoviral vectors in crude lysates were plaque-purified using limiting dilutions and agar overlays, and Ad vector clones were analyzed for presence and expression of the transgene. Positive clones were amplified for large-scale production using PER.C6 cells in 48 triple-layer 3 × 175 cm^2^ flasks. Viruses were purified by standard two-step CsCl gradient ultracentrifugation and subsequently desalted and formulated by three consecutive dialysis steps into Tris-HCl (pH 8.0) containing 2.5% glycerol. Purified Ad vectors were stored as single use aliquots at −80 °C. Virus particle titers were determined by anion-exchange HPLC based on described procedures [8]. Infectivity was assessed by TCID50 using 911 cells [9].

Adenovirus-mediated Ebola GP expression was assessed by infection of A549 cells followed by analysis of culture lysates on Western blot. The identity of the purified vectors was confirmed by PCR.

Expression vectors p1012, pGP, and pΔTM and point mutants contain a CMV enhancer promoter that has been described [2,10]. The pΔTM contains a deletion from amino acids 651 to 676 and was created by digesting with BspMI/Klenow, and then fusing to TGA. The resulting plasmid also contained four extra amino acids (aa) at the C terminus (MAAS). HEK293 cells and the derivative T-Ag-expressing 293T cell line were cultured in DMEM supplemented with 10% fetal bovine serum (GIBCO). Transfections to measure protein expression and cell rounding were performed in HEK293 cells with 2 μg of DNA per well of a six-well plate using calcium phosphate (Invitrogen) according to the manufacturer's instructions. Protein expression in cell supernatants or lysates was evaluated by SDS-PAGE followed by Western blot with a GP-specific polyclonal antibody kindly provided by A. Sanchez (Centers for Disease Control, Atlanta, Georgia, United States). Comparison of secreted and cellular expression levels was measured from 0.6% and 1%, respectively, of total sample volumes.

### Animal Study and Safety

Cynomolgus macaques *(Macaca fascicularis),* aged 3–5 y and weighing 2–3 kg, obtained from Covance (Princeton, New Jersey, United States), were used for immunization and challenge experiments. Each treatment group contained three animals unless stated otherwise. To prevent unnecessary use of animals, only one unvaccinated control was included in each study, but cumulatively over the study period, there were 29 such controls using the same virus seed stock, administered by the same route at the same dose, lethal for all animals with a mean time to death of 6.4 d. The monkeys, housed singly, were anesthetized with ketamine to obtain blood specimens and to administer vaccines. In conducting this research, the investigators adhered to the 1996 version of the animal care and usage guide prepared by the Institute of Laboratory Animal Resources [11]. The facilities are fully accredited by the Association for Assessment and Accreditation of Laboratory Animal Care International and the animals received regular enrichment. Before Ebola virus challenge and to the end of each experiment, the animals were maintained in the Maximum Containment Laboratory (BSL-4) and fed and checked daily.

### Macaque Immunization and Challenge

Cynomolgus macaques were injected intramuscularly with 1.0 ml of an equal mixture of immunogens at the doses indicated. Viral challenge was performed by inoculation of animals in the left or right caudal thigh with 0.5 ml of viral stock that contained a target dose of ˜1,000 plaque-forming units (pfu) ZEBOV at 4 wk after the initial immunization. No adverse effects of the adenovirus vaccination were observed acutely. The Ebola virus stock used in this study was originally obtained from a fatally infected human from the former Zaire in 1995 [12]. Collection of serum and blood for viral load and ELISA titers was performed as previously described [2]. Surviving animals were followed for at least 4 wk postchallenge.

### Flow Cytometry and Antibodies

Transfected cells were collected after incubation with PBS containing 3 mM EDTA and incubated with control Ig or rabbit anti-sGP/GP serum (generously provided by Dr. A. Sanchez, CDC) for 30 min on ice. The cells were washed twice with ice-cold PBS containing 2.5% fetal bovine serum, incubated with FITC- or PE-conjugated secondary antibodies (Jackson ImmunoResearch Laboratories, West Grove, Pennsylvania, United States; and Sigma-Aldrich, St. Louis, Missouri, United States, respectively) for 30 min on ice, followed by washing. Analysis was conducted using a Becton-Dickinson four-color Calibur flow cytometer and FlowJo analysis software (Tree Star, Ashland, Oregon, United States).

### ELISA

Nunc-Immuno Maxisorp plates (Nunc, Rochester, New York, United States) were coated with 100 μl/well of 10 μg/ml lectin from Galanthus nivalis (Sigma-Aldrich) in PBS and incubated overnight at 4 °C. All further incubations were carried out at room temperature. Plates were then blocked for 2 h in PBS containing 10% fetal calf serum and then washed twice with 0.2% Tween 20 (Sigma-Aldrich) in PBS. Ebola GP was obtained from the supernatants of HEK293 cells transfected with the mammalian expression plasmid Ebola GP(ΔTM) and was added to the plates at a concentration of 0.8–1.3 mg/ml total protein in 100 μl/well. Plates were then washed six times with PBS containing 0.2% Tween 20. Test sera were diluted in PBS containing 0.2% Tween 20 and 1% fetal calf serum and allowed to react with the Ag-coated wells for 60 min. After washing plates six times, goat anti-human IgG (H+L; Chemicon, Temecula, California, United States) conjugated to horseradish peroxidase was used as a detection antibody. Bound IgG was detected by Sigma Fast *O*-phenylenediamine dihydrochloride tablet sets (Sigma-Aldrich), and the optical density was determined. A panel of normal sera was run each time the assay was performed.

### Neutralizing Antibody Analysis

Ebola GP(Z) pseudotyped lentiviral virions were produced as previously described [13]. Briefly, 293T cells were plated at a density of 2 × 10^6^ per 10-cm diameter tissue culture dish and transfected the next day by calcium phosphate reagent (Invitrogen) with pCMVΔR8.2, pHR′CMV-Luc, and CMV/R Ebola GP(Z) plasmid DNA. Cells were transfected overnight, washed, and replenished with fresh medium. Supernatants containing pseudotyped virus were harvested 48 h later, filtered through a 0.45-μm pore-size syringe filter, and stored in aliquots at −80 °C. Neutralization assays were performed on HUVECs (Cambrex, East Rutherford, New Jersey, United States; #CC-2517) plated in a 24-well plate 1 d prior to infection. Virus stocks titrated to the 90% infectious dose were incubated at 37 °C for 1 h in the presence of serum from immunized cynomolgus macaques. The culture media was removed from the cells and replaced with the virus/serum media in the presence of polybrene (Sigma-Aldrich, #107689) at a final concentration of 5 μg/ml. At 72 h postinfection, cells were lysed and assayed by the Luciferase Assay System (Promega, Madison, Wisconsin, United States; #E1501/E1531). Luciferase activity was determined using a Veritas Microplate Luminometer from Turner Biosystems (Sunnyvale, California, United States).

### Intracellular Cytokine Analysis

Peripheral blood mononuclear cells were isolated from cynomolgus macaque whole blood samples by separation over Ficoll. Approximately 1 × 10^6^ cells were stimulated in 200 μl of RPMI medium (GIBCO) for 6 h at 37 °C with anti-CD28 and anti-CD49d antibodies [14], brefeldin A, and either DMSO or a pool of 15-mer peptides spanning the ZEBOV GP (Mayinga strain) open reading frame of the full length virion-bound form. There was a total of 167 15-mers overlapping by 11 spanning the entire Ebola glycoprotein at a final concentration of 2.5 μg/ml. Cells were fixed and permeabilized with FACS Lyse (Becton Dickinson, Palo Alto, California, United States) supplemented with 0.05% Tween 20, and stained with a mixture of fluorescence-conjugated antibodies against lineage markers (CD3, CD4, CD8) and TNF. Samples were run on a FACS Calibur or FACS Aria flow cytometer and analyzed using the software, FlowJo. Positive gating for lymphocytes using forward versus side scatter was followed by CD3^+^/CD8^−^ and CD3^+^/CD4^−^ gating, and specific populations were further defined by anti-CD4 and anti-CD8 positivity, respectively. Cytokine-positive cells were defined as a percentage within these individual lymphocyte subsets and at least 200,000 events were analyzed for each sample.

## Results

To develop an optimal Ebola vaccine using rAd vectors, we first analyzed mutant forms of GP in which the transmembrane domain had been removed. Although we have previously reported that deletion of the mucin domain eliminates cytotoxicity [15], this deletion removes nearly 200 aa, eliminating many potential T and B cell epitopes. Previous data suggested that the in vitro cytopathic effects of GP may be mediated at or near the cell surface and require transmembrane anchoring of the protein [16–18]. An alternative approach to the elimination of the GP-induced cytopathic effects was therefore explored by removal of the 26 aa putative transmembrane and cytoplasmic domains.

### Diminished Immune Protection of a Mutant GP Lacking a Transmembrane Anchor Domain

**Figure 1:**
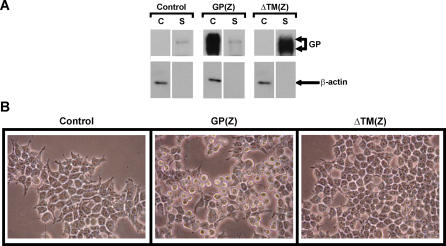
Elimination of GP Cytopathic Effects and Expression of Transmembrane-Deleted Protein (A) Expression of GP(ΔTM) in HEK293 cells. Ebola GP proteins from supernatants and cell lysates were visualized by SDS-PAGE and Western blot using a polyclonal antibody against Ebola GP. (B) Elimination of cell rounding by GPΔTM. HEK293 cells were transfected with a plasmid that encoded vector control, Ebola GP, or Ebola GPΔTM. Cell monolayers were visualized under phase contrast using a Nikon 40× objective and photographed at 24 h posttransfection.

**Figure 2:**
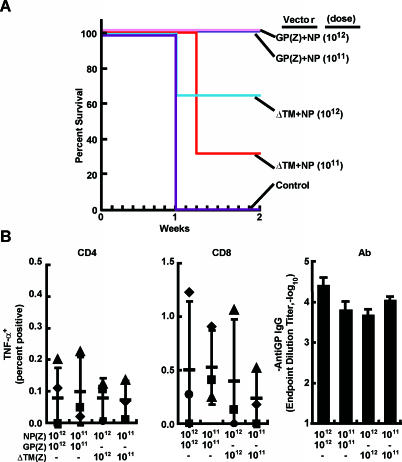
Comparative Efficacy of GP and GPΔTM for Protection against Ebola Virus Challenge (A) Kaplan-Meier survival curve of cynomolgus macaques, immunized as indicated, and challenged with 1,000 pfu of Zaire ebolavirus (strain Kikwit) 1 mo post immunization. The *x*-axis indicates weeks postchallenge. Different immunization groups had *n* = 3, except for the GP(Z) + NP (10^12^) group (*n* = 4), and control (*n* = 1). (B) Immune responses in immunized animals. Left and middle graphs: Intracellular flow cytometry was performed to quantify TNF-α production from Ebola-specific CD4^+^ or CD8^+^ lymphocytes, respectively, from animals immunized as indicated (the 10^12^ group contains only three CD8 points due to technical error in one sample). Immune responses were measured at 3 wk postimmunization. Circles, diamonds, squares, and triangles indicate responses for individual animals. Horizontal line indicates the average of individual responses in the immunization group. Results represent the percent cytokine-positive in the gated lymphocyte group, and background stimulation (DMSO alone) has been subtracted from each sample. ELISA titers of Ebola GP-specific antibodies in sera of vaccinated animals collected at week 3 postimmunization (right graph). ELISA results represent endpoint dilution titers determined by optical density as described in Methods.

GP protein was readily detected in the supernatants of cells transfected with the transmembrane-deleted vector ΔTM(Z), confirming its secretion, in contrast to supernatants from cells transfected with the wild-type GP(Z) (Figure 1A). Furthermore, synthesis of the two previously defined forms of GP, generated by posttranslational processing [19,20], was readily detected at comparable levels. Deletion of the transmembrane domain eliminated GP-induced cytopathicity in transfected HEK293 cells in contrast to wild-type GP (Figure 1B), but total ΔTM expression was equivalent to wild-type protein levels (Figure 1A). To determine whether the ΔTM mutant of the ZEBOV could protect against infectious Ebola challenge, cynomolgus macaques were immunized with rAd vectors encoding NP and either ΔTM(Z) or GP(Z). Immunization with GP(Z) + NP protected all animals vaccinated with either 10^11^ or 10^12^ adenoviral particles and challenged with 1,000 pfu of ZEBOV 28 d later (Figure 2A). In contrast, survival frequencies decreased in animals receiving the ΔTM(Z) vaccine. In the group vaccinated with 10^12^ adenoviral particles, protective immunity was decreased by 33% and, at 10^11^, by 66%, indicating a substantial decrease in efficacy in animals vaccinated with ΔTM + NP versus GP + NP (*p* < 0.05). In a separate experiment (unpublished data), 10^11^ particles of ΔTM alone failed to protect against infection. Analysis of cell-mediated immune responses showed that GP-specific CD4^+^ and CD8^+^ T-cell responses were present in the majority of animals by 3 wk postimmunization (Figure 2B, left and middle panels, respectively) and correlated well with NP-specific cellular responses (unpublished data). Antigen-specific cellular responses measured by intracellular cytokine (tumor necrosis factor-α [TNF-α]) secretion were indistinguishable between GP(Z)- and ΔTM(Z)-vaccinated animals. Similarly, humoral immune responses measured by anti-Ebola GP ELISA IgG titers were comparable in all vaccinated animals (Figure 2B, right graph). Neutralizing antibody titers were low, and were absent in some surviving animals (unpublished data). These results suggested that deletion of the GP transmembrane domain reduces vaccine efficacy, with no readily apparent correlates of protection.

### Definition of Minimal Protective Vaccine Dose for Protection with a rAd Vaccine Encoding GP and NP

**Figure 3:**
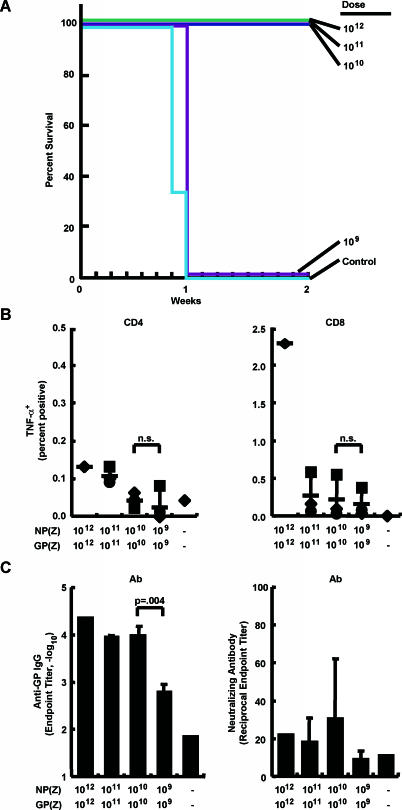
Determination of Lowest Vaccine Dose for Immune Protection against Ebola Virus Challenge by Adenoviral Vector GP/NP Vaccine (A) Kaplan-Meier survival curve of macaques: Immunization was with rAd vectors expressing GP(Z) and NP(Z) (10^9^–10^11^, three animals per group; control and 10^12^, one animal each), and challenge was performed with the Kikwit strain of Zaire ebolavirus as in Figure 2A. (B) Immune responses in immunized animals. Intracellular flow cytometry was performed to quantify TNF-α production from Ebola-specific CD4^+^ (left graph) or CD8^+^ (right graph) lymphocytes, respectively, from animals immunized as indicated. Immune responses were measured at 3 wk postimmunization. Circles, diamonds, and squares indicate responses for individual animals. Horizontal line indicates the average of individual responses in the immunization group. Results represent the percent cytokine positive in the gated lymphocyte group and background stimulation (DMSO alone) has been subtracted from each sample (*p*-values obtained using unpaired Student's *t*-test; n.s., not significant). (C) Antibody responses in immunized animals. Anti-GP ELISA titers (left graph) and serum neutralizing antibody responses (right graph) were measured as described in Methods.

In the previous experiment, a log decrease in dose of the GP(Z) + NP vaccine was protective, in comparison to previous studies using 10^12^ rAd particles. To establish the lowest dose of adenoviral vectors that would afford protection against Ebola infection, a dose-response analysis was performed. Animals were immunized with rAd vectors encoding GP(Z) and NP at increasing doses from 10^9^ to 10^12^ particles per animal. Survival was 100% in all groups receiving a dose of 10^10^ or greater, whereas challenge infection was uniformly lethal in the 10^9^ dose group (Figure 3A). Ebola virus isolation from plasma by plaque assay on Vero cells was negative for all surviving animals (unpublished data). Plasma could in theory contain factors such as immunoglobulin or cytokines that could impede virus replication. While other methods, such as RT-PCR, could potentially be used to detect such virus, a negative signal with this assay is not conclusive of sterilizing immunity, and no clear correlation between viremia and mortality has been established in nonhuman primate models of Ebola virus infection. Survival was therefore used as the study endpoint in these experiments. Prechallenge CD4^+^ T cell responses for TNF-α were unremarkable, as reported previously for immunized cynomolgus macaques (Figure 3B). CD8^+^ T cell responses were similar across vaccine dose groups, except for the higher responder immunized at 10^12^ rAd particles. Antigen-specific IgG was also generated in immunized animals, and the levels were equivalent among animals in the groups that survived Ebola virus challenge (Figure 3C, left graph). However, there was a difference of more than one log (*p* = 0.004) in IgG levels between survivors immunized at 10^10^ particles (9,600 ± 6,000) and fatalities immunized at 10^9^ rAd particles (600 ± 300), suggesting that such levels may correlate with protection for this immunization regimen. In contrast, neutralizing antibody titers against GP did not differ significantly between survivors and fatalities (Figure 3C, right graph), suggesting that ELISA IgG titers may be a stronger predictor of protective immunity for this vaccine. These results indicated that the threshold for immune protection lies at about 10^10^ rAd particles. Therefore, subsequent experiments were carried out using this dose to increase sensitivity to detect differences in antigenic strength between various immunogens.

### Identification of a GP Point Mutant with Diminished In Vitro Cytotoxicity That Confers Effective Immune Protection

**Figure 4:**
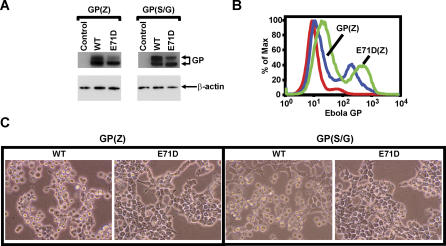
Elimination of GP Cytopathic Effects with Single Point Mutation (A) Expression of point mutants in HEK293 cells. Ebola GP proteins from supernatants and cell lysates were visualized by SDS-PAGE and Western blot using a polyclonal antibody against Ebola GP. (B) Reactivity of point mutants with a conformation-dependent antibody. HEK293 cells were transfected with a control plasmid (red line) or plasmids expressing wild-type (GP[Z], blue line), or mutant (E71D[Z], green line) proteins. 18 h posttransfection, cells were harvested and stained with a GP-specific antibody, and cell surface GP expression was analyzed by flow cytometry. (C) Elimination of cell rounding by amino acid substitution at position 71. HEK293 cells were transfected with a plasmid that encoded vector control, wild-type Ebola glycoprotein from Zaire (GP[Z]) or Sudan/Gulu (GP[S/G]), or their respective point mutations (E71D[Z] and E71D[S/G]). Cell monolayers were visualized under phase contrast using a Nikon 40× objective and photographed at 24 h posttransfection.

We sought to identify other mutants of GP that do not exhibit cytopathic effects yet retain native antigenic structures when expressed in vitro. Relatively conserved regions of GP were identified, and point mutations were systematically introduced. GP proteins bearing single-amino acid changes were screened for decreased induction of cell rounding but wild-type levels of expression and reactivity with conformation-dependent antibodies. Substitution of aspartic for glutamic acid at position 71 (i.e., E71D) in Ebola GP from the Zaire or Sudan/Gulu species (E71D[Z] or E71D[S/G], respectively) abolished the cell-rounding phenotype in transfected HEK293 cells, but did not alter protein expression or reactivity with antibodies whose binding properties are sensitive to changes in protein conformation (Figure 4).

**Figure 5:**
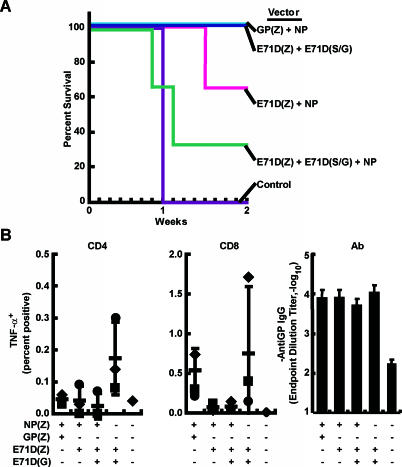
Comparative Efficacy of Wild-Type and Point Mutant Glycoprotein Vaccines against Lethal Ebola Virus Challenge (A) Kaplan-Meier survival curve of macaques: Immunization and challenge were performed with the Kikwit strain ebolavirus as in Figure 2A. (B) Immune responses in immunized animals. Left and middle graphs: Intracellular flow cytometry was performed to quantify TNF-α production from Ebola-specific CD4^+^ or CD8^+^ lymphocytes, respectively, from animals immunized as indicated. Immune responses were measured at 3 wk postimmunization. Circles, diamonds, and squares indicate responses for individual animals. Horizontal line indicates average of individual responses in the immunization group. Results represent the percent cytokine positive in the gated lymphocyte group, and background stimulation (DMSO alone) has been subtracted from each sample. ELISA titers of Ebola GP-specific antibodies in sera of vaccinated animals collected at week 3 postimmunization (right graph). ELISA results represent endpoint dilution titers determined by optical density as described in Methods.

The E71D mutants were evaluated for their ability to induce protective immunity alone or in combination with NP, compared with the wild-type GP/NP immunogen. When E71D from Zaire and Sudan/Gulu were combined with NP, survival of cynomolgus macaques immunized in these groups was diminished by 33% and 66%, respectively (Figure 5A). In contrast, complete protection was achieved in animals immunized with E71D(Z) and E71D(S/G), as it was in animals receiving wild-type GP(Z) + NP. Ebola GP-specific responses in T lymphocytes detected by intracellular staining of TNF-α did not show statistically significant differences in the CD4^+^ population between different immunization groups (Figure 5B, left graph). Similarly, individual differences in the CD8^+^ response did not correlate with survival, although there was a trend toward diminished survival in groups with lower antigen-specific CD8^+^ cellular responses (Figure 5B, middle graph). Antigen-specific ELISA IgG, which correlated well with survival in the dose-ranging experiment, was also stimulated in all immunized animals (Figure 5B, right graph). The results of this experiment illustrate that NP may not be necessary for protective immunity against Ebola infection and that it may diminish protection when combined with modified GP immunogens at the lower limits of protective vaccine doses.

## Discussion

Ebola virus outbreaks are associated with high lethality due to the absence of treatment options or a licensed vaccine. Both DNA priming with rAd vector boosting and rAd alone can confer protection to lethal challenge in an animal model that closely parallels human disease [21]. The rAd vector vaccine conferred protection in an accelerated vaccine regimen in a nonhuman primate species [3]. Although in vitro cytopathicity has been observed by overexpression of Ebola GP (one of the vaccine components), we have not seen toxicity in animals vaccinated by vectors expressing Ebola GP (unpublished data). However, because this hypothetical complication has been raised, we sought to modify GP to eliminate in vitro cytopathicity yet retain antigenic properties that are necessary for protective immunity. Here, the efficacies of different forms of GP were evaluated using doses at the threshold of protection in the accelerated vaccination model. We have identified a vaccine with decreased in vitro cytopathicity that retained immunogenicity necessary to protect against Ebola infection.

We find that alternative forms of GP confer differential immune protection. Deletion of the GP transmembrane domain abolished cytopathic effects in transfected HEK293 cells, but the corresponding ΔTM(Z) vaccine was less efficacious than wild-type GP(Z) in protecting the cynomolgus macaques against infection. Although cellular and humoral immune responses were indistinguishable between groups receiving the different immunogen forms, the inherent variability in quantitating the responses in outbred macaques may obscure our ability to identify immune responses responsible for higher survival. Alternatively, ΔTM(Z) may differ from wild-type GP(Z) in antigenic qualities that are not captured by measurements of total antigen-specific IgG or intracellular cytokine responses stimulated by a broad peptide pool. For example, the transmembrane-deleted protein is secreted and likely shows conformational differences from the membrane-anchored protein. Subsequent modifications of the glycoprotein to retain membrane attachment and a more native envelope structure yielded a mutant, E71D, with reduced in vitro cytopathicity. Recently, it was suggested that this region of GP contributes to viral receptor binding [22]. It is noteworthy that the envelope glycoprotein cytopathicity of other viruses such as HIV is linked to receptor binding and fusion [23], raising the possibility that Ebola GP shares similar properties.

Ongoing outbreaks of both Ebola and Marburg viruses illustrate the importance of developing a filovirus vaccine for human use. This report shows that protective immunity against Ebola infection is achieved in nonhuman primates by the generation of antigen-specific immune responses to a single protein, GP, which has been modified to eliminate in vitro cytopathic effects. Since the vaccine will be licensed initially against one agent, protective immunity was evaluated against challenge with this species, Zaire ebolavirus. Future studies will address whether this vaccine can protect against other Ebola species, such as SEBOV. Preliminary studies suggest that protection can be observed in animals receiving the bivalent vaccine that survive ZEBOV challenge when subsequently exposed to the Gulu strain of SEBOV (unpublished data). However, complete analysis of the question of cross-strain protection will require additional studies that fall beyond the scope of the present study.

In a dose-ranging experiment with rAd expressing GP and NP, immune protection correlated well with GP-specific ELISA IgG titers. This observation is consistent with early Ebola vaccine studies performed using a gene-based GP and NP vaccine in guinea pigs. In the current study there was also a trend toward increased survival in animals with higher CD8^+^ T cell responses when vaccines containing different antigen combinations were evaluated for protective efficacy. In combination, these findings suggest that the GP-specific humoral immune response provides a correlate of immunity; however, it is likely that the T cell response contributes to protection, and the Ig response may be a reflection of T cell help. Further improvements in the sensitivity of the T cell assay, as well as immune depletion studies, may provide further insight into this question.

Ebola virus protective immunity has also been achieved more recently using replication competent vesicular stomatitis virus vectors [24], supporting the concept that vaccination for Ebola can successfully protect against disease mortality. While this vaccine merits further investigation, and replication-competent viruses have contributed to many effective vaccines, concerns remain about live viruses because the amount of viral replication cannot be controlled, and their environmental effects are uncertain. Such considerations may pose challenges to development of vesicular stomatitis virus-based vaccines for human use. The recent recommendation that the live-attenuated polio vaccine, a relatively safe vaccine, no longer be used in the United States [25], and the inactivated virus be used instead, exemplifies these challenges. In contrast, the rAd vector vaccine is nonreplicating and can be manufactured to high yields, and safety data exist for this platform. Here we show that immunity follows a single injection with 10^10^ rAd particles, a dose that is two orders of magnitude lower than previously reported for this single-modality vaccine. Such doses of rAd vectors have proved to be well tolerated and immunogenic for other recombinant genes in vivo and can be evaluated for the vectors reported here, alone or in DNA prime/rAd boost combinations. One theoretical concern regarding a rAd vaccine is the possibility that preexisting immunity to natural Ad5 infection, occurring in up to 50% of the U.S. population, may affect vaccine efficacy. Recent preliminary results from HIV vaccine trials suggest that antigen-specific immune responses can be readily elicited by rAd5 vectors even in study participants with prior immunity to adenovirus type 5, especially when vaccine doses higher than 10^9^ are used (unpublished data). Given that Ebola GP is more immunogenic than HIV Env, we would expect that significant immune responses can be generated to the present vaccine; future clinical trials will resolve this question. In addition, DNA priming can be used prior to rAd boost to overcome Ad immunity and has also been shown to be effective in preventing Ebola infection [2]. In case Ad5 immunity should become problematic, several alternative rAd platforms, involving Ad35 and other exotic serotypes, are under development. Preexisting immunity to Ad35 is quite low (∼10%–15%) worldwide [26], and the same principles reported in this paper would apply to the development of such vaccine candidates.

Immunization with 10^10^ rAd particles of E71D(Z)+E71D(S/G) was effective against infectious challenge with ZEBOV. These data provide evidence that protection does not require the inclusion of the NP gene in the vaccine, and document that protective immunity can be conferred by responses to the GP antigen alone. Indeed, diminished survival was observed only in vaccine groups containing this gene, raising the possibility that under some conditions inclusion of NP may dilute protective immune responses. However, the significance of this result is limited by the relatively small animal numbers used here. It is noteworthy that elimination of NP from the vaccine and dose reductions to 10^10^ rAd particles do not diminish protection, and simplify the vaccine for future development in human trials. A first-generation Ebola DNA vaccine is currently under evaluation in phase I clinical studies, and the rAd component is currently being manufactured as a clinical grade preparation that is undergoing safety studies and will be tested in Phase I trials pending regulatory approvals. After the completion of initial studies testing individual DNA and rAd vaccine components, these candidates will be evaluated in prime boost combination. Vaccine safety and immunogenicity relative to the nonhuman primate challenge model will guide assessments regarding its utility as a preventive vaccine in humans.

## Supporting Information

Alternative Language Abstract S1 Translation of the Abstract into French (23 KB DOC)

## Abbreviations

aa: amino acid
Ad5: adenovirus type 5
GP: glycoprotein
NP: nucleoprotein
pfu: plaque-forming unit
rAd: replication-defective adenoviral (vector)
SEBOV: Sudan ebolavirus
TNF: tumor necrosis factor
ZEBOV: Zaire ebolavirus
