# Supplementary material for: Immune Protection of Nonhuman Primates against Ebola Virus with Single Low-Dose Adenovirus Vectors Encoding Modified GPs
Source: PLoS Med. 2006 May 16;3(6):e177. doi: 10.1371/journal.pmed.0030177 (PMC1459482; doi:10.1371/journal.pmed.0030177)
Supplement: Supplementary file 1 [file pmed.0030177.sd001.doc]

**RÉSUMÉ**

**Antécédents :** En l’absence de thérapies efficaces contre les infections par le virus Ebola, le développement d’un vaccin devient une stratégie importante en vue d’endiguer les déclarations de ce virus. Une immunisation au moyen de vecteurs d’ADN et/ou de vecteurs adénoviraux défectifs pour la réplication (rAd) encodant la glycoprotéine (GP) et la nucléoprotéine (NP) du virus Ebola s’est révélée conférer, dans le passé, une immunité protectrice spécifique chez des primates non humains. La GP peut exercer des effets cytopathiques sur les cellules transfectées *in vitro* et de nombreuses formes de GP ont été identifiées dans la nature, ce qui soulève la question de savoir laquelle d’entre elles serait optimale pour un vaccin humain.

**Méthodes et résultats :** Pour aborder cette question, nous avons exploré l’efficacité de GP mutantes en provenance de nombreuses souches du virus Ebola, avec effet cytopathogène *in vitro* réduit et analysé leurs effets protecteurs sur le modèle à défi de primate, avec ou sans NP. L’élimination du domaine transmembranaire de GP a éliminé l’effet pathogène *in vitro* mais a réduit son efficacité protectrice d’au moins un ordre de grandeur. Par contre, une mutation ponctuelle a été identifiée supprimant cet effet cytopathogène, tout en conservant le caractère immunogène et en conférant une protection immunitaire en l’absence de NP. La dose minimale efficace de rAd a été établie à 10^10^ particules, deux logarithmes plus bas que ce qui a été utilisé auparavant.

**Conclusion :** L’expression seule de GP spécifiques vectorisées par rAd est suffisante pour conférer une protection contre le défi mortel dans un modèle primate non humain pertinent. L’élimination de la NP du vaccin et des réductions de dose à 10^10^ particules de rAd ne diminuent pas la protection et simplifient le vaccin, fournissant la base nécessaire à une sélection de candidat-vaccin humain.
